# Successively accelerated ionic wind with integrated dielectric-barrier-discharge plasma actuator for low-voltage operation

**DOI:** 10.1038/s41598-019-42284-w

**Published:** 2019-04-09

**Authors:** Shintaro Sato, Haruki Furukawa, Atsushi Komuro, Masayuki Takahashi, Naofumi Ohnishi

**Affiliations:** 1 Department of Aerospace Engineering, Tohoku University, Sendai, 980-8579 Japan; 2 Department of Electrical Engineering, Tohoku University, Sendai, 980-8579 Japan

## Abstract

Electrohydrodynamic (EHD) force is used for active control of fluid motion and for the generation of propulsive thrust by inducing ionic wind with no moving parts. We propose a method of successively generating and accelerating ionic wind induced by surface dielectric-barrier-discharge (DBD), referred to as a DBD plasma actuator with multiple electrodes. A conventional method fails to generate unidirectional ionic wind, due to the generation of a counter ionic-wind with the multiple electrodes DBD plasma actuator. However, unidirectional ionic wind can be obtained by designing an applied voltage waveform and electrode arrangement suitable for the unidirectional EHD force generation. Our results demonstrate that mutually enhanced EHD force is generated by using the multiple electrodes without generating counter ionic-wind and highlights the importance of controlling the dielectric surface charge to generate the strong ionic wind. The proposed method can induce strong ionic wind without a high-voltage power supply, which is typically expensive and heavy, and is suitable for equipping small unmanned aerial vehicles with a DBD plasma actuator for a drastic improvement in the aerodynamic performance.

## Introduction

**Figure 1:**
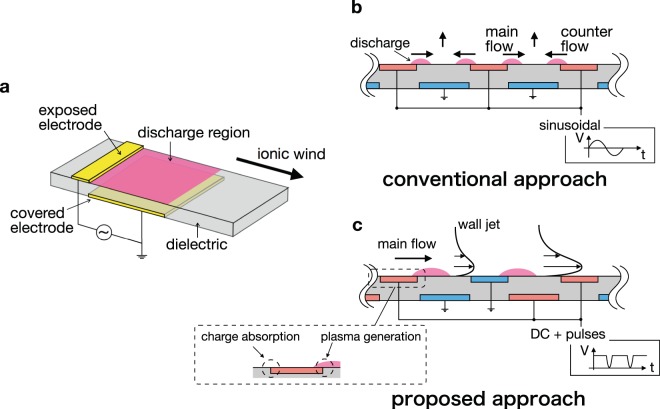
Schematic views of a DBD plasma actuator and method of integrating the actuator modules. (**a**) A single DBD plasma actuator composed of an exposed electrode, covered electrode, and a dielectric. Discharge occurs above the covered electrode when high voltage is applied to electrodes, generating ionic wind. (**b**) The conventional approach to integrating the actuator modules fails to monotonically accelerate the ionic wind, due to the generation of counter ionic-wind. (**c**) The proposed approach accelerates ionic wind by designing the electrode arrangement and the applied voltage such that mutually enhanced (rather than mutually degraded) EHD force is generated. In this arrangement, the downstream and upstream sides of the exposed electrode edge generate plasma and absorb the dielectric surface charge, respectively.

Birds, insects, fish, and swimming mammals perceive fluid motion and actively control its flow in order to generate lift efficiently and to enhance locomotive performance^1–3^. However, it is highly challenging for humans to control the fluid motion. Active flow control has the potential to drastically improve the hydrodynamic performance such as suppression of the separating flow and drag reduction, contributing to a reduction of the CO_2_ emissions from cars and airplanes^4^ and power required to pump fluid in pipes^5^. Most active flow-control devices currently available control flow by changing the shape of the device using some mechanical moving parts (e.g., leading edge slats or trailing edge flaps to control the flow around an airfoil). These mechanical flow-control devices can modify the flow and enhance the performance of fluid machinery; however, there are some inherent disadvantages such as complicated components, non-negligible weight increase, low responsiveness. Active flow control techniques using the electrohydrodynamic (EHD) effect are one of the most promising candidates for next-generation flow-control devices, owing to their advantages over existing flow control devices –i.e., because they have no moving parts and offer high-speed responsiveness^6^. The EHD effect induces an ionic wind^7,8^, which modifies the flow field around the fluid machinery and generates propulsive force^9^. Active flow-control devices that use EHD force are referred to as plasma actuators, and they have been studied extensively in the last twenty years. In particular, plasma actuators using dielectric barrier discharge (DBD)^10^ are preferred over actuators using DC corona discharge^11^. This is because the transition to an arc discharge, which leads to degradation of the actuator due to heat and renders the power supply unstable, is prevented by the dielectric barrier in the DBD plasma actuators. A single DBD plasma actuator typically consists of two electrodes and one dielectric, and generates non-thermal plasma around the exposed electrode (Fig. 1a). Several researchers have demonstrated that the DBD plasma actuators suppress flow separation^12,13^ and control the boundary layer transition^14,15^. However, flow control using a DBD plasma actuator at high-speed airflow is confronted by the difficulty of producing sufficient ionic wind for the flow control.

In order to improve the performance of DBD plasma actuators, optimization studies in view of electrical and fluid properties have been conducted^16^. A straightforward method of enhancing the induced flow velocity is to increase the applied voltage amplitude. Some previous studies show that the induced flow velocity increases monotonically with increasing voltage but then becomes saturated at high voltage levels^17,18^. The maximum induced velocity reaches 6 m/s when the peak-to-peak value of the applied voltage is 50 kV^19^. A critical drawback to increasing the amplitude of applied voltage is that a high-voltage power supply is needed, and it is usually expensive and heavy on aircraft. A previous study demonstrated the flow control effect in low-velocity flow (typically the airflow speed is less than 30 m/s)^6^. This result indicates that the DBD plasma actuator is feasible for controlling flow, provided that the cruising speed is less than 108 km/h, as with small unmanned aerial vehicles (UAVs)^20^. However, equipping small UAVs with high-voltage power supplies hinders their practical applications. In addition, considerable isolation distance between the DBD plasma actuator and the other electronic devices is required in order to avoid unexpected discharge when high voltage is applied to the DBD plasma actuator. This requirement is also a drawback from the point of view of practical applications.

Another method of enhancing EHD force involves using several DBD plasma actuators in series. A previous experimental study observed the additional acceleration of ionic wind and a maximum velocity of approximately 7 m/s, measured using four actuator modules (a single actuator module is composed of two electrodes, i.e., a single DBD plasma actuator) with a voltage peak-to-peak value of 40 kV and a frequency of 1 kHz^19^. However, there must be a gap between each actuator module, because counter ionic-wind is induced when successive actuator modules are close together or when high voltage is applied, degrading the performance of the integrated DBD plasma actuator. This is known as the cross-talk phenomenon^21^. The need for a gap restricts the integrated density of the actuator modules and impedes efficient acceleration of ionic wind. Counter ionic-wind is generated as a result of the discharge between a covered electrode and the exposed electrode of adjacent modules as shown in Fig. 1b. This counter wind collides with the main flow and defects wind in the vertical direction to the dielectric surface when there is no gap between each actuator module, as in the case of so-called plasma synthetic jet actuators^22^. The generation of the counter ionic-wind due to the cross-talk phenomenon is undesirable in view of the multiplied acceleration of ionic wind by successive actuator modules.

In this study, we propose a multi-electrode DBD plasma actuator that mutually enhances the EHD force between successive actuator modules, rather than generating counter ionic-wind. The proposed actuator couples a particular applied voltage waveform with an electrode arrangement that is suitable for generating unidirectional EHD force. Figure 1c shows the proposed electrode arrangement and the applied voltage waveform. Our approach focuses on the charge on the dielectric surface and on controlling the surface charge cycle, insofar as the surface charge plays a dominant role in the DBD^23^. The applied voltage waveform is DC voltage combined with repetitive nanosecond pulses rather than sinusoidal voltage. On the one hand, DC voltage is desirable for accelerating the charged particles, although the electric field generated by constant voltage is likely to be affected by electric field screening due to the charge on the dielectric surface in the DBD. On the other hand, the nanosecond pulse, which has a large voltage-slope, is suitable for generating charged particles to neutralize the surface charge. This waveform is apt for generating and accelerating charged particles^24^. In the DBD plasma actuator driven by DC voltage combined with repetitive pulses, the charge cycle of the dielectric surface consists of two strokes: positive charging by the DC voltage, and a neutralizing positive charge by the nanosecond pulses^23^.

The electrode arrangement in the proposed multi-electrode DBD plasma actuator differs from conventional approaches. In a conventional multi-electrode DBD plasma actuator, all of the exposed electrodes are connected to a power supply, and all of the covered electrodes are grounded. In our arrangement, the exposed electrodes are connected to the power supply and ground alternately, and the covered electrodes are arranged as shown in Fig. 1c. With this arrangement, counter ionic-wind is prevented because there is no potential difference between a covered electrode and the exposed electrode of the adjacent module. Consequently, no discharge is generated. Moreover, the upstream side of the adjacent exposed electrode absorbs the surface charge and prevent electric field screening, enhancing the EHD force rather than degrading it.

The potential benefit of our system is that an EHD force comparable to that of high-voltage-operation single-module actuators can be generated with low-voltage-operation highly densely integrated actuator modules because the driving voltage can be reduced by scaling down the single module and the discharge region can be extended by increasing the number of modules. The highly integrated DBD plasma actuator can actively control the airflow over the whole surface of the body, whereas the control surface is limited to the discharge region generated by two electrodes in a conventional DBD plasma actuator. Our strategy for improving the performance of DBD plasma actuators is based on simple dynamics of surface discharge, and it has the potential to dramatically enhance the ionic wind generation with low-voltage operation. Thus far, no study has investigated the mutual enhancement effect in a multi-electrode DBD plasma actuator with a focus on the surface charge behavior. We performed a numerical simulation of the discharge process and experimentally confirmed the mutually enhancing effect by measuring the thrust induced by the discharge and the induced flow velocity.

## Results

### Numerical simulation of the discharge process

**Figure 2:**
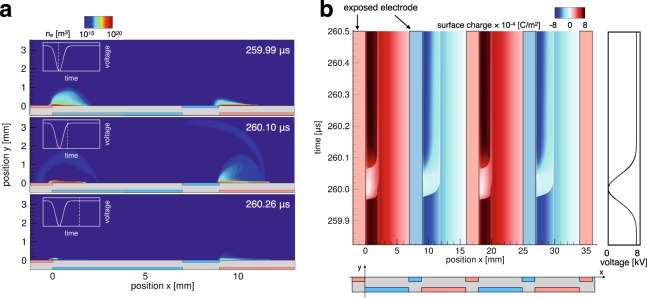
(**a**) Spatial distributions of electron number density during the nanosecond-pulse phase and DC phase. In the voltage falling phase, diffusive and streamer discharges occur above the grounded and high-voltage covered electrodes, respectively. By contrast, in the voltage rising phase, streamer and diffusive discharges occur above each electrode. The discharge stops after pulse superposition owing to surface charge accumulation. (**b**) Time history of the surface charge for the multi-electrode configuration. The phase of the applied voltage is shown to the right of the figure. Positive charging and neutralizing strokes form above the grounded electrode whereas negative charging and neutralizing strokes form above the high-voltage electrode around the downstream side of the exposed electrodes. In addition, the magnitude of the surface charge continuously reduces around the upstream side of the exposed electrodes.

To confirm EHD force enhancement using the multielectrode DBD plasma actuator with our strategy, a numerical simulation of the discharge process was conducted using the plasma fluid approximation based on previous studies^23,25,26^. A detailed description of the simulation method can be found in the Methods section. The model employed in this study agreed well with previous experimental and numerical studies^23,27–29^ and can reproduce the dielectric surface charge behavior^25^. We conducted a numerical simulation for the nine-electrode case. The applied voltage waveform was 8-kV DC voltage combined with repetitive pulses of 25 kHz and 100-ns FWHM. The peak value of the pulse was −8 kV such that it was instantaneously grounded. Discharge forms repetitively with the same frequency as the pulse superposition. The spatial distribution of the electron number density is shown in Fig. 2a. In the voltage falling phase, diffusive discharge occurs above the grounded covered electrode, whereas the streamer-like discharge occurs above the high-voltage covered electrode. By contrast, in the voltage rising phase, streamer-like and diffusive discharges are observed above the grounded and high-voltage covered electrodes, respectively. These discharge structures are the same as those of discharges triggered by the nonbiased NS pulses as reported in previous experimental^30^ and numerical^31^ studies, indicating that the DC voltage has only a small effect on the discharge structure. The electron number density during the pulsed discharge is in the order of 10^20^ m^−3^ (up to 10^21^ m^−3^ at the streamer head); this value is typical of atmospheric discharge^32^. The discharge disappears through diffusion and recombination during the DC phase because the electric field is gradually screened by the accumulated dielectric surface charge.

A two-stroke charge cycle forms at each actuator module owing to pulse superposition (Fig. 2b). Positive charging and neutralizing strokes, which are the same strokes described in a previous numerical simulation^23^, are observed above the grounded covered electrode. By contrast, negative charging and neutralizing strokes form above the high-voltage covered electrode. As a result, positively and negatively charged areas are alternately obtained. In addition, the magnitude of the surface charge decreases around the upstream side of each exposed electrode owing to charge absorption by the exposed electrode. Note that the surface charge downstream is constantly charged and screens the electric field in the two-electrode configuration case^23,33^ because the area neutralized by the pulse superposition is limited near the downstream side of the exposed electrode, as shown in Fig. 2b.

**Figure 3:**
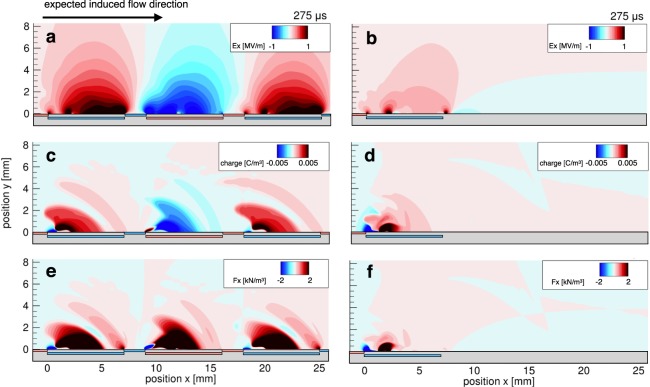
Distributions of (**a**,**b**) the *x*-component of the electric field (*E*_*x*_), (**c**,**d**) the space charge density, and (**e**,**f**) the EHD force parallel to the dielectric surface for nine- and two-electrode cases. An 8-kV DC voltage combining 25-kHz negative 8-kV pulses was applied in the discharge simulation. Both *E*_*x*_ and space charge are positive above the grounded covered electrode, whereas *E*_*x*_ and space charge are negative above the high-voltage covered electrode in the nine-electrode case. Positive EHD force is generated above each covered electrode. The magnitude of *E*_*x*_, the space charge, and the EHD force in the nine-electrode case is higher than in the two-electrode case because the exposed electrode on the downstream side absorbs the surface charge, enhancing the EHD force.

Figure 3a shows the distribution of the *x*-component of the electric field (*E*_*x*_) 15 *μ*s-after the pulse superposition. Positive and negative *E*_*x*_ regions were generated above the covered electrode by the grounded and high-voltage electrodes, respectively. A wider high-electric-field region was generated at *x* = 0–7 mm than in the two-electrode case (i.e., with the single-module DBD plasma actuator), as shown in Fig. 3a,b. This indicates that the exposed electrode of the adjacent actuator module reinforces the electric field above the covered electrode owing to charge absorption by the exposed electrode, as shown in Fig. 2b, when the distance between successive actuator modules is short.

**Figure 4:**
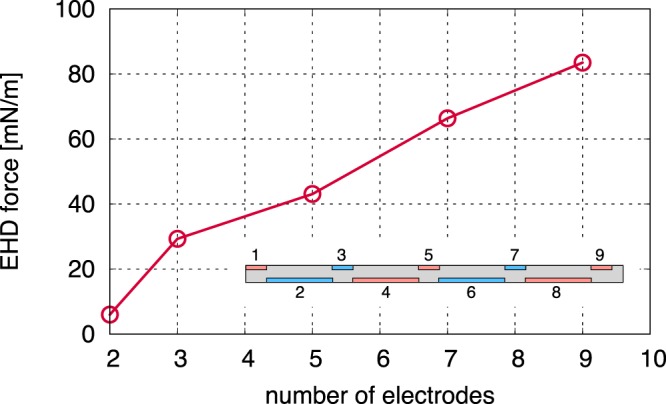
Time-averaged and space-integrated EHD force parallel to the dielectric surface as a function of the number of electrodes. The method of counting electrodes is described in the figure. The increment of the EHD force from two electrodes to three electrodes is caused by the absorption of the surface charge. The integrated EHD force linearly increases with an increase in the number of electrodes beyond three.

The positively and negatively charged regions appear alternately after the occurrence of discharge as shown in Fig. 3c. In addition, charged particles are generated at the downstream edge of the exposed electrode, and no discharge occurs at the upstream side of the exposed electrode edge, because the electric field strength is not large enough to ignite the discharge due to the same potentials between the covered electrode and the exposed electrode of the adjacent actuator module. The space charge density in the single-module DBD plasma actuator is lower than that in the multi-electrode plasma actuator case, because the charged particles cannot travel downstream due to the electric field screening effect (Fig. 3d). The distribution of EHD force parallel to the dielectric is shown in Fig. 3e. Positive EHD force is consequently generated above each covered electrode, because the positively charged particle in the positive *E*_*x*_ region and negatively charged particle in the negative *E*_*x*_ also generate positive EHD force. The simulation result indicates that in the proposed multi-electrode DBD plasma actuator, counter ionic-wind, which is caused by negative EHD force, is not generated insofar as discharge ignition from the upstream edge of the exposed electrode is prevented. Moreover, considerable EHD force is generated at *x* = 0–7 mm compared to the single-module DBD plasma actuator (Fig. 3f), indicating that the proposed multi-electrode DBD plasma actuator mutually enhances the EHD force of actuator modules rather than degrading it (as reported in previous study^19^). Small EHD force parallel to the dielectric surface is generated in the two-electrode case (5 mN/m), whereas the EHD force in the three-electrode case is 26 mN/m (Fig. 4). This increment is the result of suppressing the accumulation of surface charge, and this was observed in a previous experimental study with a similar electrode configuration^34^. The EHD force increases almost linearly when increasing the number of electrodes beyond three, indicating that EHD force can be enhanced by increasing the number of electrodes instead of increasing the amplitude of the applied voltage. Note that the proposed multi-electrode DBD plasma actuator has no gap between each actuator module, and that EHD force increases almost linearly with the number of electrodes. By contrast, EHD force does not increase linearly in the conventional multi-electrode DBD plasma actuator, which includes a gap between each actuator module to avoid the generation of counter ionic-wind^35^.

### Discharge characteristics

**Figure 5:**
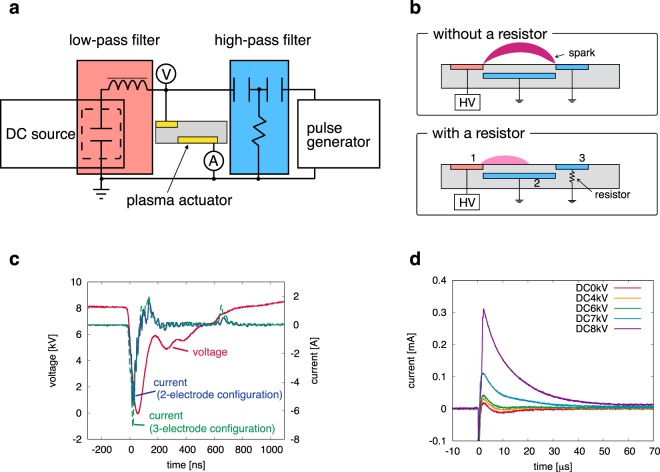
(**a**) Schematic view of the experimental setup. The DC source is connected to the DBD plasma actuator via a low-pass filter, while the pulse generator is connected via a high-pass filter to prevent interference between power supplies. (**b**) Conceptual diagram of the three-electrode DBD plasma actuator with a current limiting resistor. The resistor (1 MΩ) is added to the exposed electrode, which plays a role in surface-charge absorption, to avoid sparks. (**c**) Typical voltage and current waveforms measured by the probes as shown in (**a**). Similar current waveforms are obtained in the two- and three-electrode configurations, except for the peak value. (**d**) Typical current waveforms passing through the resistor, as presented in (**b**). The peak value increases with an increase in DC voltage, indicating that more positive ions are collected and absorbed by the third electrode.

An experimental investigation of the discharge characteristics was carried out with the two- and three-electrode configurations in order to experimentally demonstrate the result obtained in the numerical simulation and reveal the effect of the exposed electrode downstream. DC voltage combined with repetitive nanosecond pulses was realized using a DC source and pulse generator, as shown in Fig. 5a. In order to prevent interference between power supplies, the DC source and the pulse generator were connected to the DBD plasma actuator via a low-pass filter and a high pass filter, respectively^36^. The power supplies were connected to the exposed electrode upstream, whereas the covered electrode and the exposed electrode downstream were connected to the ground. A detailed description of the experimental setup is found in the Methods section.

A spark, which can damage the DBD plasma actuator and render the power supply unstable, occurs between the exposed electrodes in the proposed electrode configuration when the DC voltage is sufficiently high to ignite, as reported in previous studies^37,38^. In this study, a current limiting resistor (1 MΩ) was inserted between the exposed electrode downstream and the ground for the purpose of stabilizing surface discharge, as shown in Fig. 5b. In the two-electrode configuration and in the three-electrode configuration with the current limiting resistor, negative and positive current pulses were observed during the falling and rising phases of the voltage, respectively (Fig. 5c). The discharge during the falling phase occurs due to the potential difference between the exposed electrode and the dielectric surface, which is positively charged before the pulse superposition due to the DC voltage. In this phase, an electric field is generated in the direction from the dielectric surface to the exposed electrode upstream. Therefore, the negatively charged particles are spread downstream, neutralizing the positively charged dielectric surface (i.e., the neutralizing phase)^23^. In the voltage rising phase, the peak amplitude of the current pulse is smaller than that in the voltage falling phase. Contrary to the falling phase, positively charged particles spread downstream because the electric potential at the exposed electrode upstream is higher than the potential at the dielectric surface during the rising phase. These characteristics of current are similar to the current waveform when the negative nanosecond-pulse without DC voltage is applied^39^, indicating that the DC component of the applied voltage has little impact on the electrical characteristics.

The peak amplitude of the current pulse during the falling phase is slightly higher in the three-electrode configuration than in the two-electrode configuration. This suggests that the electric field is enhanced due to the exposed electrode downstream. A similar trend was observed in nanosecond pulsed sliding discharge, which had a similar electrode configuration^40^. Figure 5d shows the time histories in the three-electrode configuration of the current passing through the resistor, derived from the voltage difference across the resistor using Ohm’s law. The current rapidly increases after the pulse superposition (the negative pulse is superposed at *t* = 0 *μ*s) and decays slowly, indicating that positively charged particles reach the exposed electrode downstream and transfer their charges to the ground via the resistor. This result clearly shows that the exposed electrode downstream prevents the accumulation of charge, which screens the electric field generated by the DC voltage. The peak value of the current at the exposed electrode downstream increases with an increase to the DC voltage. This is because the amount of positive ions arriving at the exposed electrode downstream increases when the DC voltage increases, due to the enhancement of the electric field. It should be noted that the decay time of the current is sufficiently short to absorb the positive ions until the successive pulse superposition under the condition of this study (the repetitive pulses frequency is up to 2000 Hz).

**Figure 6:**
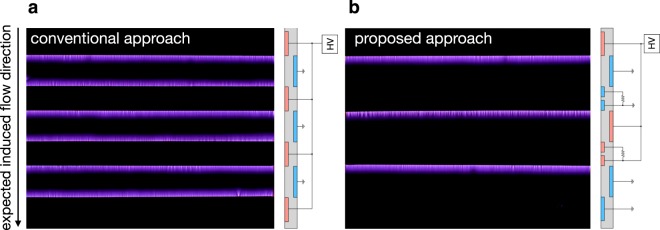
Discharge photographs for (**a**) conventional and (**b**) proposed electrode arrangements using seven electrodes when applying 8-kV DC voltage combining 2000-Hz negative 8-kV pulses. The electrode arrangement is schematically shown on the right-hand side of each photograph. 200 pulses were applied during the camera’s exposure. Discharge occurs at the both sides of the exposed electrode for (**a**). By contrast, discharge spreads from upstream side of the exposed electrode for (**b**), preventing unexpected discharge, which generates counter ionic-wind.

In order to discuss the discharge structure, a photograph of the discharge in the seven-electrode configuration was taken using the conventional configuration with 2000-Hz pulses applied and an optical gate of 0.1 sec (Fig. 6a). The discharge spreads from both sides of each exposed electrode because there is a large potential difference between the exposed electrode and the covered electrode at both sides, as discussed above. This discharge structure generates counter ionic-wind, which decelerates the main flow^19^. Contrary to the conventional approach, the discharge occurs and spreads from the downstream side of each exposed electrode with the proposed approach, as shown in Fig. 6b. Note that an exposed electrode was divided into two electrodes in this experiment. One electrode was connected to the power supply or ground directly, and the other was connected via a resistor to limit the current. The upstream side of the exposed electrode plays a role in charge absorption and requires a resistor to prevent sparks. Conversely, the downstream side of the exposed electrode plays a role in the ionization of air and cannot ignite the discharge when a high resistive material is connected to the electrode due to the current limitation. The gap between each divided electrodes is 2 mm. Discharge does not occur in this gap because the potential difference between the divided electrodes is small. The distance of 2 mm between each actuator module is quite small compared to conventional multistage DBD plasma actuators (e.g., 20 mm in ref.^19^). The peak voltage of the third electrode is 300 V, as shown in Fig. 5d (0.3 mA × 1.0 MΩ). The breakdown threshold of air at atmospheric pressure is around 30 kV/cm^41^. Therefore, this distance can be shortened to the order of 0.1 mm in our system. It is interesting to note that the discharge structure above the high-voltage covered electrode is filamentary, whereas the structure above the grounded covered electrode is relatively diffusive. This is caused by the different type of the discharges; cathode- and anode-directed discharges occur above the high-voltage and grounded covered electrodes, respectively.

### Thrust measurement and successive acceleration of ionic wind

**Figure 7:**
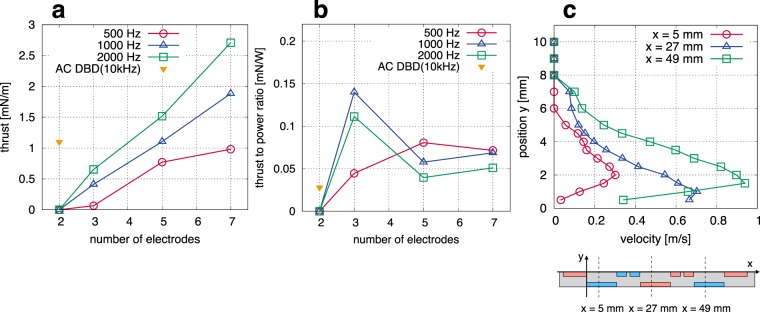
(**a**) Thrust generated by the proposed DBD plasma actuator as a function of the number of electrodes when applying 8-kV DC voltage combining negative 8-kV pulses with a frequency of 500, 1000, and 2000 Hz. This is consistent with the numerical simulation results, and more thrust was obtained at a higher pulse repetition. The thrust produced by a typical AC DBD plasma actuator under similar experimental conditions (except for frequency)^42^ is also plotted for comparison. (**b**) Thrust to power ratio as a function of the number of electrodes. The efficiency of our method is higher than that of a typical AC DBD plasma actuator when we use three or more electrodes. (**c**) Stream-wise velocity profiles for the proposed method with a repetitive pulse frequency of 2000 Hz. No apparent flow was obtained for with the conventional method, whereas a wall jet formed and accelerated using the proposed approach.

The thrust generated by the surface discharge and the induced flow velocity were measured in quiescent air to characterize the performance of the proposed multi-electrode DBD plasma actuator. The effect of the number of electrodes on the thrust parallel to the dielectric surface was measured when 8-kV DC voltage was applied combined with negative 8-kV pulses at a repetitive pulses frequency of 500, 1000, 2000 Hz (Fig. 7a). For the sake of comparison, the method for counting electrodes is the same as shown in Fig. 4 (i.e., the two separated electrodes are counted as one electrode). Although no thrust was measured in the two-electrode case in this study, the thrust increased almost linearly with an increased number of electrodes at all frequencies, consistent with the results of the numerical simulation. This indicates that the degradation of the thrust-generation performance due to the cross-talk phenomenon is suppressed in the proposed approach. The increment of the thrust from the two-electrode to three-electrode cases is caused by the enhancement of the electric field downstream due to the absorption of the surface charge by the exposed electrode. Therefore, this result clearly shows the mutual enhancement (rather than degradation) of the thrust generation between successive actuator modules.

In addition, the thrust increases with an increase to the repetitive pulse frequency, because the generation rate of charged particles increases. The positive EHD force is mainly generated during the dielectric charging phase^23^. The total EHD force corresponds to the sum of the EHD force produced by each nanosecond pulse superposition when the period of the repetitive pulses is longer than the time scale of the positive or negative charging phase. Thus, EHD force generation could start to saturate when the nanosecond pulse is superposed before the dielectric surface is fully charged. However, this time scale is of the order of 0.1 *μ*s, as shown in Fig. 2b, which corresponds to 10 MHz and is much shorter than the period of repetitive pulses. EHD force generation could also start to saturate when the period of the repetitive pulses becomes shorter than the time required for charge absorption at the upstream side of the exposed electrode. The time constant of charge absorption is roughly 20 *μ*s (i.e., 50 kHz), as shown in Fig. 6d. This time constant is also much shorter than the pulse repetition period under our experimental conditions and can be easily reduced by using a low-resistance load. Therefore, large thrust can be obtained by increasing the repetitive pulse frequency.

The proposed method produces the same order of thrust as a typical AC DBD plasma actuator under similar experimental conditions^42^. The result of the AC DBD plasma actuator is obtained with the same peak-to-peak voltage but with frequency of 10 kHz. This study mainly aims to provide a highly integrated multi-electrode plasma actuator without generating counter ionic wind. Although the optimization of the electrode gap and the shape of the nanosecond pulse for enhancement of the EHD force generated by the single-module actuator should be addressed, the study of EHD force enhancement in the single-module actuator is beyond the scope of this study and will be performed as a future work. The advantage of our approach over the conventional multi-electrode AC DBD plasma actuator is that each module actuator can be densely arranged, resulting in efficient additional acceleration of the ionic wind. In light of the thrust to power ratio, the efficiency of our approach is the same as or higher than that of a typical AC DBD plasma actuator when we use three or more electrodes, as shown in Fig. 7b. The power deposited to air is calculated as described in the previous experimental study^43^, which uses a nanosecond pulse voltage. The efficiency of our proposed method approaches around 0.06 mN/W with an increase in the number of electrodes because both the thrust and the power increase when the number of actuator modules increases.

Our single module resembles a conventional three-electrode plasma actuator^44^. Although a performance comparison should be conducted, it will be made as a future work because the present study focuses on the multistage plasma actuator and not on the single-module performance. The main difference between other three-electrode actuators and our single-module actuator is that the DC voltage is applied to the downstream exposed electrode in the conventional method whereas it is superposed on the nanosecond pulses and is applied to the upstream exposed electrode in our method. The idea of using a DC voltage resembles that of the conventional three-electrode configuration in view of the acceleration of charged particles. However, the previous three-electrode plasma actuator was used for a single module and cannot be arranged densely. By contrast, the distance between each module can be made quite small in our system.

In order to discuss the successive acceleration effect of ionic wind, stream-wise velocity profiles at *x* = 5, 27, and 49 mm for the conventional and proposed methods were measured with a Pitot tube when applying 8-kV DC voltage combining negative 8-kV pulses with the repetitive pulses frequency of 2000 Hz. The origin of the coordinate is at the downstream side of the first exposed electrode. In the conventional approach, no apparent flow was induced at any location in this experimental condition, due to the interaction between the main ionic wind and the counter ionic-wind. By contrast, the generation of a unidirectional wall jet generation and its successive acceleration obtained with the proposed approach (Fig. 7c). The peak value of the velocity was 0.3 m/s at *x* = 5 mm and accelerated to around 1 m/s at *x* = 49 mm. This result suggests ionic wind can be enhanced by increasing the number of electrodes without increasing the applied voltage amplitude. Note that larger induced velocity was reported in previous studies. However, these studies used a high-voltage amplitude (e.g., around 7 m/s was measured using four modules with peak-to-peak value of 40 kV^19^). Large induced velocity will be obtained when the applied voltage increases in our system; however, increasing the applied voltage is beyond the scope of this study. Our objective in this paper is to propose a high-density multi-electrode plasma actuator that can generate unidirectional ionic wind and not to prove that our system produces larger induced velocity than other DBD plasma actuators.

## Discussion

Our results demonstrated that the multi-electrode DBD plasma actuator proposed in this study generated mutually enhanced EHD force and successively accelerated the ionic wind, whereas no apparent flow was induced with the conventional approach due to the cross-talk phenomenon, as reported in previous studies. The applied voltage waveform is DC voltage combined with repetitive nanosecond pulses forming a two-stroke cycle of the surface charge. The DC component plays a role in accelerating the charged particle, while the nanosecond pulses generate charged particles. The proposed electrode arrangement prevents the generation of counter ionic-wind because there is no potential difference between the covered electrode and the downstream side of the exposed electrode. Moreover, the upstream side of the exposed electrode absorbs the surface charge and reinforces the electric field, enhancing EHD force rather than degrading the other actuator modules. Our strategy for strong ionic wind generation differs completely from that of the conventional approach, which requires very high-voltage, making it unsuitable for industrial applications. The results presented in this paper suggest that the applied voltage can be lowered by making the dielectric thinner to the limit of the breakdown voltage. In air, Paschen’s low reveals a minimal breakdown voltage of approximately 350 V in a uniform electric field. The EHD force can be increased by increasing the number of electrodes instead of increasing the amplitude of the applied voltage. The actuator module is miniaturized using microelectronic fabrication technology^45^. The reduction of the driving voltage and further integration of actuator modules will be performed in future work. Moreover, the thrust can be controlled with DC-bias voltage and pulse repetition frequency independently by using DC-biased repetitive pulses to better control thrust generation.

Although the performance of the DBD plasma actuator is impractical for high-speed airflow control, as noted by other researchers, the feasibility of controlling the flow field around an object flying at low speeds (e.g., a small UAV) has been experimentally demonstrated. Therefore, the issues that remain for practical applications of the DBD plasma actuator include reducing the driving voltage, though little attention has been paid to this. Our method of integrating DBD plasma actuator modules does not require a high-voltage power supply, which is typically expensive and heavy. Our results suggest that the highly integrated multi-electrode DBD plasma actuator generates strong ionic wind without increasing the voltage amplitude, and that it is thus able to drastically improve in the aerodynamic performance of a small UAV. The highly integrated DBD plasma actuator is also applicable to an active flow control device using EHD force for various types of fluid machinery, because its low-voltage operation makes it easy to use the active flow control device and reduces the cost of introducing a DBD plasma actuator system (in particular, its power supply).

## Methods

### Numerical methods

The simulation of the discharge process was conducted using the plasma fluid approximation^23,25,26^. The governing equations consist of continuity equations for charged particles and Poisson’s equation for electric potential. The numerical flux was evaluated with the Scharfetter–Gummel shceme^46^, and the Euler implicit method was employed as the time integration method for the continuity equations. Poisson’s equation was solved with the semi-implicit technique^47^. The transport parameters of the charged particles were determined based on the local electric field and obtained using the BOLSIG + electron Boltzmann equation solver^48^. The charged particle temperatures were assumed to be constant as previous numerical study of nanosecond-pulse-driven plasma actuator^49^. The electron temperature and ion temperatures were 1 eV and 300 K, respectively. The numerical methods used in this study were described previously in refs^23,28,29^.

### Simulation condition

The ambient gas was assumed to be dry air. The simulation domain was 40.0 × 10.5 mm^2^ and divided into 1940 × 336 non-uniform grid points. The dielectric thickness was 0.5 mm, and the relative permittivity of the dielectric was 5 as an epoxy resin. The width of the exposed and covered electrode was 2 mm and 7 mm, respectively. The applied voltage was represented as follows^23^:

1 $$V(\tau )={V}_{dc}-{V}_{dc}\,\exp \{-\,\frac{{(\tau -T/2)}^{2}}{2{\sigma }^{2}}\}\,(0 < \tau \le T),$$

where *τ* = *t*–*nT* is a wave phase, *V*_*dc*_ is a DC voltage, *n* is a non-negative integer, and *T* is the period of repetitive pulses. The e-folding *σ* is a function of the full width at half maximum (FWHM): $\sigma =\mathrm{FWHM}/(2\sqrt{2\,\mathrm{ln}\,2})$. At the beginning of the simulation, the following smooth step-like voltage is applied:

2 $$V(t)={V}_{dc}\{0.5\,\tanh (\frac{t-a}{b})+0.5\},$$

where the parameters *a* and *b* were set to 1.0 ns and 0.3 ns, respectively. All the simulations terminated at 300 *μ*s in this study.

### Experimental setup

A schematic view of the experimental setup is described in Fig. 5a. A custom-designed DC source and a pulse generator were connected to the DBD plasma actuator via filters. The low-pass filter consisted of inductors and a capacitor, and the high-pass filter comprised capacitors and a resistor. The waveforms of voltage and current were measured using a high-voltage probe (PMK PHV4002-3) and a current probe (Pearson model 2877), respectively. The electrode was 0.05 mm thick copper tape. The width of the exposed and covered electrodes was 10 mm and 12 mm, respectively. The exposed electrode was divided into two electrodes, as shown in Fig. 6b, such that the width of the electrode was 4 mm with a gap of 2 mm between each electrode. The dielectric was made with four layers of 0.08 mm thick polyimide tapes (i.e., a total thickness of 0.32 mm). The span-wise length of the DBD plasma actuator was 100 mm. The discharge photographs were taken with a digital camera (Nikon D5500) with a lens (Tamron SP AF 60 mm F/2 Di II LD [IF] MACRO 1:1) and an exposure time of 0.1 sec (200 pulses), F/4, and ISO 3200.

### Thrust measurement

The measurement of the time-averaged thrust produced by the DBD plasma actuators characterizes the performance and is simple to conduct. The thrust generated by the DBD plasma actuator was measured using an electronic balance (A&D FX-300i), which is commonly used to estimate thrust^50,51^. The DBD plasma actuator was mounted on the balance with an acrylic stand so that it induced an upward flow. The generated thrust can be determined by measuring the downward reaction force of the actuator. This thrust corresponds to the EHD force minus the viscous drag at the wall^16^. The balance was placed in a Faraday cage in order to prevent electromagnetic interference. The time-averaged thrust was estimated during 4-sec operation, which is sufficient to become a steady state.

### Measuring the induced ionic wind velocity

The ionic-wind velocity was measured using a Pitot tube made of a glass tube (0.5 mm inside diameter and 0.8 mm outside diameter), rather than stainless steel, in order to prevent electrical interaction with the discharge. The glass tube was connected to a differential pressure sensor (Sensirion SPD31). The induced velocity *u* was obtained as follows:

3 $$u=\sqrt{\frac{2{\rm{\Delta }}p}{\rho }},$$

where Δ*p* is the pressure difference, and *ρ* is the air density. We assumed that the air density at the measurement positions is not affected by the discharge because the heated region is confined to the immediate vicinity of the exposed electrode edge and rapidly cools to room temperature^49^ We corrected the velocity profiles as a consequence of wall proximity^52^. The time-averaged velocity was estimated during 4-sec operation, which is sufficient to become a steady state.
